# Has the frequency of complicated appendicitis changed in children in the first year of the COVID-19 pandemic?

**DOI:** 10.1186/s43159-022-00235-7

**Published:** 2023-01-11

**Authors:** Tülin Öztaş, Salim Bilici, Ahmet Dursun

**Affiliations:** Department of Pediatric Surgery, University of Health Sciences Diyarbakır Gazi Yaşargil Training and Research Hospital, Diyarbakır, Turkey

**Keywords:** Appendicitis, Complicated appendicitis, COVID-19 pandemic, Postoperative complications

## Abstract

**Background:**

The COVID-19 pandemic period suggests that the rate of complications may have increased in patients requiring surgical treatment due to the fact that they could not come to the hospital at the onset of the symptom. This study aims to evaluate the difference in the frequency of complicated appendicitis and postoperative complications in the COVID-19 pandemic.

Patients included those who underwent appendectomy in 1 year before the COVID-19 pandemic and in the first year of the pandemic. The patients were categorized into two groups: pre-pandemic and pandemic periods. Clinical and histopathology results were compared between the pre-pandemic and pandemic periods.

**Results:**

A total of 407 patients were included in the study, 207 of whom were included during the pre-pandemic and 200 of whom during the pandemic period. The mean time to hospital admission after the onset of symptoms was 1.3 ± 0.9 days, pre-pandemic, and 1.4 ± 0.8 days during the pandemic group. In the pre-pandemic group, 0.4% intrabdominal abscess developed and 37.5% complicated appendicitis was detected. In the pandemic group, it was found that there were 1% abscess, 0.5% wound infection, 0.5% brid ileus, and 31.9% complicated appendicitis. The pre-pandemic group length of hospitalization was 2.4 ± 0.8 days, and the pandemic was 2.1 ± 0.9 days There was no difference between pre-pandemic and pandemic groups in terms of age, gender, white blood cell count, duration of symptoms, postoperative complications and frequency of complicated appendicitis, and duration of hospitalization.

**Conclusions:**

In the first year of the COVID-19 pandemic, we found that the rate of complicated appendicitis and postoperative complications were not different from pre-pandemic.

## Background

The COVID-19 pandemic process, which started in December 2019 and continues, caused disruption in the routine functioning of life. In order to control the spread of the disease, restrictions such as quarantine and curfew were applied, elective operations were postponed, and only emergency operations were performed [1]. During the pandemic period, the number of patients admitted to the hospital for reasons such as fear of contact with patients with COVID-19 and difficulty in transportation, especially in rural areas, decreased [2, 3].

Appendicitis is the most common disease requiring emergency surgery in children. Prolongation of the time between the onset of symptoms and admission to the hospital for 48 h or more increases the risk of developing complications such as ischemia, gangrene, and perforation in the appendix [1, 4]. The COVID-19 pandemic period suggests that the rate of complications may have increased in patients requiring surgical treatment due to the fact that they could not come to the hospital at the onset of the symptom. Although it has been reported in some published studies that the frequency of perforated appendicitis increased in the first months of the pandemic [5–11], some studies reported that the frequency of complicated appendicitis did not change [3, 12]. The objective of the study is to evaluate whether there is a difference in the frequency of complicated appendicitis and postoperative complications in the first year of the COVID-19 pandemic period compared to a year ago.

## Methods

In this study, hospital records of patients who were operated in the pediatric surgery clinic of our hospital with the diagnosis of appendicitis in 1 year before the COVID-19 pandemic and in the first year of the pandemic were retrospectively analyzed. Patients who underwent appendectomy between March 2019 and March 2021 were included in the study. Patients who underwent incidental appendectomy and had incomplete data were excluded from the study. The present study was approved by the Institutional Ethics Committee.

The patients were categorized into two groups: pre-pandemic and pandemic periods. The patients who underwent appendectomy between March 2019 and February 2020 were named the pre-pandemic. The pandemic period consisted of those who underwent appendectomy between March 2020 and February 2021.

Demographic data of the patients, laboratory tests, time between the onset of symptoms and admission to the hospital, operation findings, histopathology results, length of hospitalization, and postoperative complications were recorded. Wound infection, abscess formation in the abdomen, and bride were evaluated as postoperative complications. According to histopathological examination, those who were diagnosed with lymphoid hyperplasia and appendix vermiformis were evaluated as negative appendicitis and those who were diagnosed with gangrenous, phlegmonous, or perforated appendicitis were considered as complicated appendicitis. Histopathology reports were classified into three groups: negative appendicitis, acute appendicitis, and complicated appendicitis.

Appendicitis frequency, white blood cell (WBC) count, duration of symptom, duration of hospitalization, postoperative complications, and histopathology results were compared between pre-pandemic and pandemic periods.

### Statistical methods

Data were analyzed using SPSS (SocialSciences software package version 22.0 Windows) software program. Categorical variables were determined as number (*n*) and percentage (%). Numerical variables with normal distribution were shown as mean ± standard deviation. Continuous variables were compared using Student’s *T* test and Mann–Whitney *U* test. *p* < 0.05 was considered statistically significant.

## Results

**Fig. 1:**
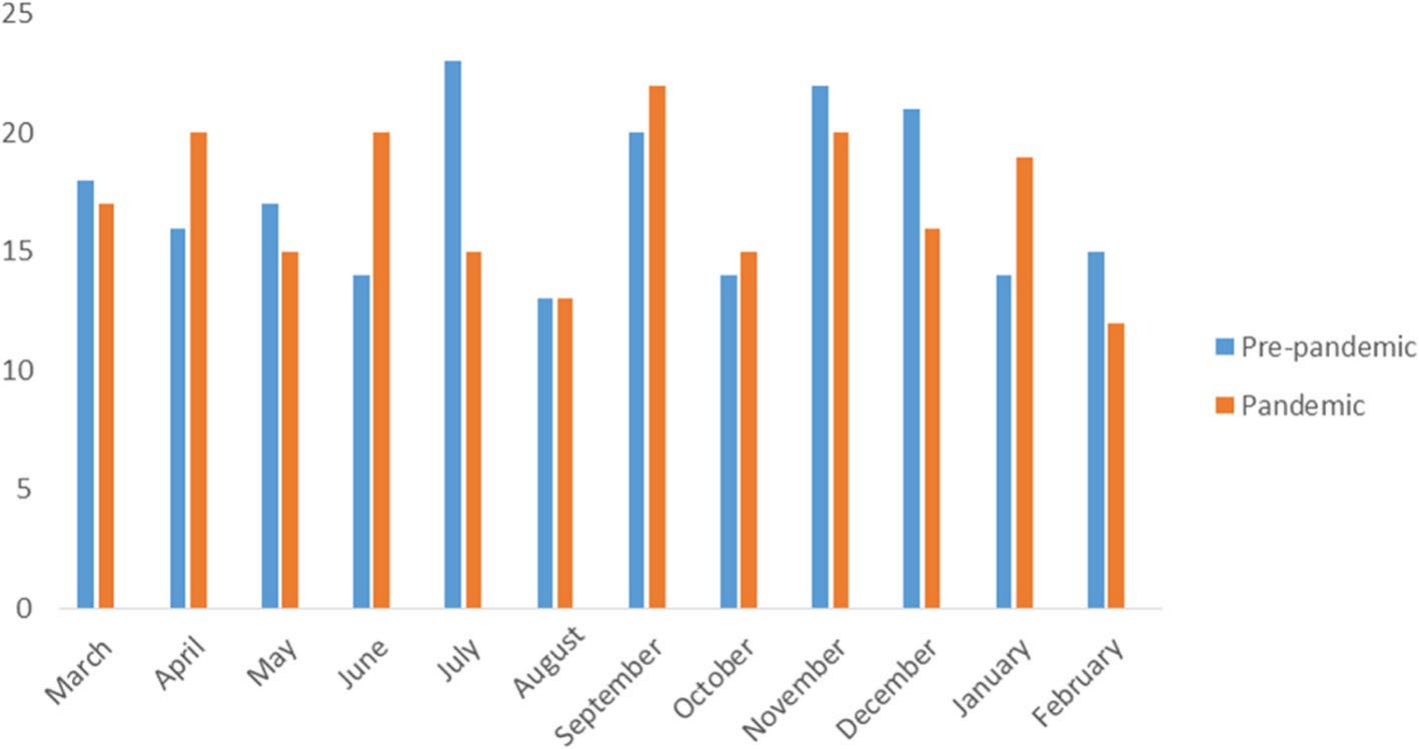
The number of appendicitis in the pre-pandemic and pandemic periods

A total of 407 patients were included in the study. The number of appendectomies by months is presented in Fig. 1.

The pre-pandemic group consisted of 207 patients, 77 girls and 130 boys. In the pandemic period, there were 200 patients, 69 of whom were girls and 131 were boys. There was no statistically significant difference between the two groups in terms of age, gender, and WBC (*p* > 0.05).

The mean time to hospital admission after the onset of symptoms was 1.3 ± 0.9 days (1–7), pre-pandemic, and 1.4 ± 0.8 days (1–6) during the pandemic period (*p* = 0.08). The pre-pandemic length of hospitalization was 2.4 ± 0.8 days (1–8), and the pandemic period was 2.1 ± 0.9 days (1–5). The duration of hospitalization in the two groups was not different (*p* = 0.06).

In the pre-pandemic group, 0.4% intrabdominal abscess developed in the postoperative period. In the pandemic period group, it was found that 1% abscess, 0.5% wound infection, and 0.5% brid ileus developed. We did not find any difference in the development of postoperative complications in the pre-pandemic and pandemic groups (*p* = 0.08).

Histopathology results were 12% negative appendicitis, 56.1% acute appendicitis, and 37.5% complicated appendicitis in the pre-pandemic. In the pandemic period, 10.5% negative appendicitis, 52% acute appendicitis, and 31.9% complicated appendicitis were detected. There was no significant difference between the two groups in terms of the frequency of complicated appendicitis (*p* = 0.43) (Table 1).

**Table 1:** Clinical and pathological characteristics of the patients in the pre-pandemic and pandemic periods

Characteristic	Pre-pandemic (*n* = 207)	Pandemic (*n* = 200)	*p* value
Mean age (years)	11.8 ± 3.2 (4–17)	11.5 ± 3.3 (1–17)	0.07
**Gender**			0.76
Female	77 (37.2)	69 (34.5)	
Male	130 (62.8)	131 (65.5)	
Mean WBC (µL)	10.6 ± 8.4 (9.3–28)	11.3 ± 7.0 (5.4–30.2)	0.27
Duration of symptoms (days)	1.3 ± 0.9 (1–7)	1.4 ± 0.8 (1–6)	0.08
Length of hospitalization(days)	2.1 ± 0.8 (1–5)	2.4 ± 0.9 (1–8)	0.06
**Pathology**			0.43
Lymphoid hyperplasia	21 (10.1)	17 (8.5)	
Appendiks vermiformis	4 (1.9)	4 (2)	
Acute appendicitis	116 (56.1)	104 (52)	
Phlegmonous appendicitis	50 (24.2)	41 (20.5)	
Gangrenous appendicitis	0 (0)	13 (6.5)	
Perforated appendicitis	16 (7.7)	21 (10.5)	
**Postoperative complications**			0.08
Abscess	1 (0.5)	2 (1)	
Bride	0 (0)	1 (0.5)	
Wound infection	0 (0)	1 (0.5)	

*WB*C White blood cell

## Discussion

The COVID-19 pandemic continues despite restrictions, protective measures, and vaccination. During the pandemic period, difficulties were experienced in the management of patients with both appendicitis and other diseases requiring urgent intervention. During the pandemic period, the number of patients diagnosed with appendicitis increased in public hospitals due to the closure of private hospitals in rural areas [13]. Montalya et al. reported in their study that parents preferred pediatric hospitals because they thought there was less risk of transmission, and the number of patients who underwent appendectomy increased by 77% during the pandemic period [14]. However, the results of our study showed that there was no difference in the frequency of appendicitis in the first year before and during the pandemic. In our study, it was concluded that the frequency of appendicitis did not change due to the fact that the parents thought that the COVID-19 virus infects children less and that there may be fewer contact with COVID-19 patients in the pediatric emergency department, so there was no decrease in the hospital admission of children with abdominal pain.

In patients with suspected appendicitis, the time between the onset of symptoms and the time of admission to the hospital was prolonged during the COVID-19 pandemic [9, 13, 15]. In the early stages of the epidemic, it was observed that patients did not go to the hospital unless they had significant symptoms due to the limitation of protective masks and personal protective equipment [1]. Some published studies have reported that there is no difference in symptom duration of patients who underwent appendectomy compared to the pre-pandemic [7, 10, 12, 14, 16]. In our study, the time between the onset of symptoms and hospital admission in the pandemic period was longer than the pre-pandemic. However, this difference was not statistically significant. Reasons such as the fear of contact with the patient with COVID-19 in the hospital, the imposition of a pandemic curfew, and difficulty in transportation may have contributed to the prolongation of the admission period.

The WBC count and C-reactive protein levels are higher in blood tests of patients with appendicitis who were operated during the pandemic period [13]. As in many studies and our study, it was observed that there was no significant difference in WBC count between patients who underwent appendectomy during the pandemic period and patients who underwent appendectomy before the pandemic [2, 10, 11, 14, 17–20]. The results of our study suggest that there is no difference in the count of WBC before and during the pandemic period, since the patients came to the hospital early before the infection progressed during the pandemic period. There was no change in the duration of hospitalization of patients who underwent appendectomy during the pandemic period [7, 9, 10, 14]. In some studies, it was found that patients were discharged as soon as possible due to reasons such as reducing the risk of postoperative nosocomial infections and the need for an empty bed during the pandemic period, and therefore, the duration of hospitalization was shortened [13, 15]. Gerall et al. stated that during the pandemic period, patients with acute appendicitis came to the hospital late, so there were more complications, and the duration of hospitalization was longer than before the pandemic [11]. Although it was not statistically significant, in our study, it was determined that the hospitalization after appendectomy was shorter than the pre-pandemic period. The thought of doctors to reduce the risk of patients and their attendants related to contact with patients with COVID-19 and families’ desire to be discharged with the same fear were effective in the early discharge of the patient in this process.

Patients and their parents do not prefer to come to the hospital due to the risk of transmission, and the doctors’ limited examination due to the fear of contact causes the patients to be diagnosed late [3]. It has been reported that appendix perforation is more common during the pandemic period [2, 10, 11, 20] and one-third of the patients are perforated [4]. Although it was not statistically significant during the pandemic period, two times the appendix perforation was detected compared to the pre-pandemic period [3, 21]. In another study, as in our study, no significant increase was observed in the frequency of complicated appendicitis during the pandemic period [1]. The fact that the period between the onset of complaints and admission to the hospital in our patients in the first year of the pandemic is similar to that before the pandemic explains the absence of an increase in the rate of complicated appendicitis.

It has been stated that complications such as complicated appendicitis, intra-abdominal abscess, and wound infection in the postoperative period are more common since patients cannot go to the hospital due to fear of COVID-19 transmission in the first period of the pandemic and only if they go to the hospital when the pain is unbearable [1]. However, in some studies, it was observed that there was no difference in the complications that developed pre-pandemic and the pandemic period [6, 7, 14, 17]. In our study, no difference was observed in the rate of development of postoperative complications in the pre-pandemic and in the pandemic. The fact that the symptom duration of the patients was similar to the pre-pandemic and therefore they were treated in the initial period of the infection explains that the rate of complications does not increase during the pandemic period.

### Limitations

The limitations of our study are as follows: a single center, retrospective. There is a need for prospective and multi-center studies. In the future, studies comparing children infected with COVID-19 between appendicitis and non-infected children may be conducted.

## Conclusions

Although some published studies reported an increase in the rate of complicated appendicitis, especially in the early stages of the pandemic, in the first year of the COVID-19 pandemic, we found that the rate of complicated appendicitis and postoperative complications were not different from pre-pandemic. Nevertheless, it should not be overlooked that families should be informed in this process to care about abdominal pain in children and to apply to health institutions in a short time.

## Data Availability

The datasets (SPSS files) used and/or analyzed during the current study are available from the corresponding author on reasonable request.

## Abbreviations

WBC: White blood cell

## References

[1] Ho SL, Lau J, Wang C-T, Cheung S-L, Wong K-F, Leung S-K (2021). Impact of coronavirus disease 2019 (COVID-19) on acute appendicitis in Hong Kong: retrospective cohort study in a local cluster hospital. Surg Pract..

[2] Finkelstein P, Picado O, Muddasani K, Wodnicki H, Mesko T, Unger S (2021). A retrospective analysis of the trends in acute appendicitis during the COVID-19 pandemic. J Laparoendosc Adv Surg Tech A.

[3] Snapiri O, Rosenberg Danziger C, Krause I, Kravarusic D, Yulevich A, Balla U (2020). Delayed diagnosis of paediatric appendicitis during the COVID-19 pandemic. Acta Paediatr.

[4] Wang AW, Prieto J, Ikeda DS, Lewis PR, Benzer EM, Van Gent JM (2021). Perforated appendicitis: an unintended consequence during the coronavirus-19 pandemic. Mil Med.

[5] Romero J, Valencia S, Guerrero A (2020). Acute appendicitis during coronavirus disease 2019 (COVID-19): changes in clinical presentation and CT findings. J Am Coll Radiol.

[6] Köhler F, Acar L, van den Berg A, Flemming S, Kastner C, Müller S (2021). Impact of the COVID-19 pandemic on appendicitis treatment in Germany-a population-based analysis. Langenbecks Arch Surg.

[7] Tankel J, Keinan A, Blich O, Koussa M, Helou B, Shay S (2020). The decreasing incidence of acute appendicitis during COVID-19: a retrospective multi-centre study. World J Surg.

[8] Ielpo B, Podda M, Pellino G, Pata F, Caruso R, Gravante G, et al. Global attitudes in the management of acute appendicitis during COVID-19 pandemic: ACIE Appy Study. Br J Surg. 2020; 8:10.1002/bjs.11999. 10.1002/bjs.11999 PMC7675377 34157090

[9] KumairaFonseca M, Trindade EN, CostaFilho OP, Nácul MP, Seabra AP (2020). Impact of COVID-19 outbreak on the emergency presentation of acute appendicitis. Am Surg.

[10] Zhou Y, Cen LS (2020). Managing acute appendicitis during the COVID-19 pandemic in Jiaxing. China World J Clin Cases.

[11] Gerall CD, DeFazio JR, Kahan AM, Fan W, Fallon EM, Middlesworth W (2020). Delayed presentation and sub-optimal outcomes of pediatric patients with acute appendicitis during the COVID-19 pandemic. J Pediatr Surg.

[12] La Pergola E, Sgrò A, Rebosio F, Vavassori D, Fava G, Codrich D (2020). Appendicitis in children in a large Italian COVID-19 pandemic area. Front Pediatr.

[13] Baral S, Chhetri RK, Thapa N (2021). Comparison of acute appendicitis before and within lockdown period in COVID-19 era: a retrospective study from rural Nepal. PLoS ONE.

[14] Montalva L, Haffreingue A, Ali L, Clariot S, Julien-Marsollier F, Ghoneimi AE (2020). The role of a pediatric tertiary care center in avoiding collateral damage for children with acute appendicitis during the COVID-19 outbreak. Pediatr Surg Int.

[15] Cano-Valderrama O, Morales X, Ferrigni CJ, Martín-Antona E, Turrado V, García A (2020). Acute care surgery during the COVID-19 pandemic in Spain: changes in volume, causes and complications. A multicentre retrospective cohort study. Int J Surg..

[16] Velayos M, Muñoz-Serrano AJ, Estefanía-Fernández K, Sarmiento Caldas MC, MoratillaLapeña L, López-Santamaría M (2020). Influence of the coronavirus 2 (SARS-Cov-2) pandemic on acute appendicitis. An Pediatr (Engl Ed).

[17] Ganesh R, Lucocq J, Ekpete NO, Ain NU, Lim SK, Alwash A (2020). Management of appendicitis during COVID-19 pandemic; short-term outcomes. Scott Med J.

[18] Fisher JC, Tomita SS, Ginsburg HB, Gordon A, Walker D, Kuenzler KA (2021). Increase in pediatric perforated appendicitis in the New York city metropolitan region at the epicenter of the COVID-19 outbreak. AnnSurg.

[19] English W, Habib Bedwani N, Doganay E, Marsden M, Muse S (2021). Suspected appendicitis and COVID-19, a change in investigation and management-a multicentre cohort study. Langenbecks Arch Surg.

[20] Meriç S, VartanogluAktokmakyan T, Tokocin M, Aktimur YE, Hacım NA, Gülcicek OB (2021). Comparative analysis of the management of acute appendicitis between the normal period and COVID-19 pandemic. Ulus Travma Acil Cerrahi Derg.

[21] Turanli S, Kiziltan G (2021). Did the COVID-19 pandemic cause a delay in the diagnosis of acute appendicitis?. World J Surg.

